# Mechatronic Anti-Collision System for Electric Wheelchairs Based on 2D LiDAR Laser Scan

**DOI:** 10.3390/s21248461

**Published:** 2021-12-18

**Authors:** Wiesław Szaj, Paweł Fudali, Wiktoria Wojnarowska, Sławomir Miechowicz

**Affiliations:** 1 Department of Physics and Medical Engineering, Faculty of Mathematics and Applied Physics, Rzeszow University of Technology, Powstańców Warszawy 6, 35-959 Rzeszow, Poland; wwojnarowska@prz.edu.pl; 2 Department of Mechanical Engineering, The Faculty of Mechanical Engineering and Aeronautics, Rzeszow University of Technology, Powstańców Warszawy 8, 35-959 Rzeszow, Poland; pfudali@prz.edu.pl (P.F.); smiechow@prz.edu.pl (S.M.); 3 Doctoral School of Engineering and Technical Sciences, Rzeszow University of Technology, Powstańców Warszawy 8, 35-959 Rzeszow, Poland

**Keywords:** electromobility, wheelchair electric security system, indoor localization, LiDAR, disabled persons, collision avoidance, autonomous vehicle

## Abstract

Electric wheelchairs make it easier for disabled and elderly people to live, move, interact, and participate in society. Moving a wheelchair in open spaces is relatively easy, but in closed and small spaces, maneuvering is difficult. Solutions to such problems for people with disabilities are applicable to a relatively small group of recipients and are mostly custom-made solutions, whose considerable cost is a significant barrier to accessibility. New technologies can provide an opportunity to improve the quality of life of people with disabilities in this aspect. Using selected elements of complex automation and control systems, cost-effective solutions can be created that facilitate the functioning of people with disabilities. This paper presents an analysis of hazards and problems when maneuvering a wheelchair in narrow passageways, as well as the authors’ solution to this problem, and the concept and assumptions of a mechatronic anti-collision system based on 2D LiDAR laser scanners. This solution is composed of a proprietary 2D rotating scanner mechanism that ensures the acquisition of 3D images of the environment around the wheelchair. Preliminary tests of this solution yielded promising results. Further research will include miniaturization of the device.

## 1. Introduction

Mobility is an essential component of physical well-being [1,2]. One of the most commonly used assistive devices to improve the personal mobility of people with disabilities is a wheelchair. Unfortunately, most disabled people lack the strength to take care of themselves manually. As a result, they require powered wheelchairs, but these are not appropriate for people with cognitive impairment, such as dementia or Alzheimer’s disease. Even for those without cognitive impairment, safely driving a wheelchair is challenging because it requires considerable skill, attention, judgment, and appropriate behavior. It requires the driver not only to be able to control the wheelchair but also to be constantly aware of his or her surroundings to avoid colliding with surrounding people, objects, and walls. Such a collision for a wheelchair user can result in injury or even a fall. Wheelchair collisions are dangerous not only for wheelchair users but also for other people. When an elderly person is hit, he or she may suffer a hip fracture, which can result in a long healing process, complications, and even death due to complications. Damage caused by the collision of the wheelchair with fixtures, that is, destruction of furniture, walls, etc., is also a significant problem. Due to these risks, it is necessary to minimize the possibility of collisions. A possible solution could be to develop an obstacle detection system.

New developments in control systems, drives, and autonomous vehicles can be applied to devices for people with disabilities to improve their safety. A significant barrier to the availability of these devices is the high price of individual components, which translates into a significant cost of the finished device. However, in industrial applications, this problem is of less importance. Solutions for the disabled are applicable to a relatively small group of customers, and in most cases, these are custom-made solutions (adjusted to the needs of an individual user). However, by using selected elements of complex automation and control systems, cost-effective solutions can be created that facilitate the functioning of people with disabilities in today’s world. Therefore, new technologies can provide an opportunity to improve the quality of life of people with disabilities.

One of the recent research directions is collision avoidance. However, research and development of vehicles and wheelchairs for people with disabilities addresses issues related not only to collision avoidance but also to improving ergonomics, seat fit, and functionality for specific dysfunctions. Wheelchair users face the accessibility problem of climbing stairs, particularly in developing countries, where many buildings lack ramps and lifts. There are commercially available stair-climbing wheelchairs and wheelchairs suitable for use on difficult terrains or unpaved roads, but their cost is beyond the means of many disabled people. To date, research suggests that the problem of climbing stairs for wheelchair users remains an ongoing problem. Some of these studies focused on designing wheelchairs with the ability to access stairs [3], while others aimed to develop a wheelchair add-on [4]. Another line of research is to develop or improve the design of a wheelchair with upright function [5,6]. Such solutions allow the user to rise to an upright position, thus facilitating daily life. There is also research on wheelchair control systems. Research in this area includes adapting the control manipulator to the specifics of the present dysfunctions, e.g., upper-limb disorders [7], and other alternative control methods, e.g., a chin- [8], facial-expression- [9], or breath-controlled manipulator [10]. There is also ongoing work on control issues using other senses, such as vision [11,12] or brainwave control [13]. There are solutions available on the commercial market for wheelchairs controlled by remote control or a mobile application. However, these solutions do not provide collision avoidance protection.

Research in the area of collision avoidance is focused, among other things, on systems that prevent collision with people or objects near the wheelchair. Various solutions for such systems can be found in the literature, and collision avoidance systems are usually related to issues such as mapping, localization, and simultaneous localization and mapping (SLAM). Approaches to dealing with these issues vary widely. Mapping consists mainly of creating a map, model, or representation of the environment using sensor data. The literature associated with the task of map building proposes two main frameworks: the metric map, which represents the environment with geometric precision, and the topological map describing the environment in a series of maps containing locations and related connections between them. Localization is a task that attempts to estimate the current position and orientation of an object in the environment. In addition to mapping and subsequent localization tasks, SLAM offers mixed solutions. This process is performed by continuously building maps and updating them, while the object simultaneously estimates its position in the model [14]. One of the recent developments of anti-collision systems is the solution presented by Xiao et al. [15], which is based on an extended Kalman filter (EKF) that combines the optical flow divergence (OFD) with inertial sensors.

The ideas and solutions used in collision avoidance systems are very diverse. Collision avoidance systems detect a dangerous collision and warn the user or directly take braking/driving action. They use different detection systems to detect the hazard. One of the main differences among these detection systems is the type of sensors used. They use radar or other types of sensors, such as lasers or cameras. The choice of collision detection system depends on the established requirements based on the user’s needs and the technical capabilities.

Some collision avoidance systems for the detection of objects around the vehicle use ultrasonic sensors [16,17,18]. These solutions are usually based on fuzzy controllers, where the control logic decides to change the direction of movement or stop the vehicle if a detected object is determined to be too close. However, ultrasonic sensors can interact with each other, which can generate unnecessary warnings. The radiation characteristics of ultrasonic detectors force them to be positioned in such a way that the detection zones do not overlap, and they often have to work sequentially. The relatively long detection time in the sequential operation mode limits the speed of the system. Research presented in an article by Dutta and Fernie [19] showed that this type of sensor has a limited ability to detect objects when the beam angle is small. This situation occurs when moving in a narrow corridor near walls. The detection of objects such as people’s lower limbs or furniture is also limited. Therefore, using an ultrasonic sensor in a collision avoidance system for wheelchairs may not be adequate.

Another type of detection system used in collision avoidance systems is stereovision systems [20,21]. The cameras used in this solution provide an image with a depth map (RGB-D). Sensors of this type include cameras that use one of two methods to determine the depth map. The first one is based on illuminating the object with structured light and determining the distance based on the distortion of the displayed pattern. The second one is based on illumination with pulsating light and determination of the phase shift between emitted and registered light returning from the object. An example of such sensors is the Kinect camera. The range of depth cameras is limited, especially in sunlight. The light emitted by the camera can be completely “covered” by sunlight. Another example of using a camera for developing a collision avoidance system was presented in work by Narayanan et al. [2], who designed a visual wall collision avoidance task around an image-based visual servoing scheme that relies on low-cost architecture that includes a single monocular camera and a haptic joystick.

Another detection method is the use of a 2D laser scanner. This solution has been applied, for example, in an autonomous wheelchair by Grewal et al. [22]. LiDAR was also used by Wang et al. to develop a mapping system that enables a robot to autonomously survey large physical structures [23]. The advantage of scanners is the accurate measurement of the distance independent of lighting conditions. The 2D LiDAR scanner can perform 360-degree measurements in one narrow plane, which, in a wheelchair anti-collision system, can provide observation of the entire environment of the disabled person.

Such scanners can provide information about the environment around the wheelchair as long as its field of view is not obscured by the user and the components of the wheelchair. This requires the scanner to be mounted above the user’s head [24]. However, this is not a very practical solution. Another way to solve the problem of obscuring part of the scanning zone is to multiply the scanners. Nevertheless, a single scanning plane limits the possibility of detecting objects at different heights. Another method of solving this problem can be the use of 3D scanners that perform scanning in several planes simultaneously. Three-dimensional scanners are characterized by a wide measurement range. These devices are dedicated to specific professional applications, which significantly affects their price. The cost of these devices is still very high compared to the price of the wheelchair in which they are to be installed. Suppose that two or three units are needed in the wheelchair; their price may exceed the value of the vehicle itself. For economic reasons, such expensive detectors can only be justified for research and development purposes. In practical applications where the group of end users is relatively small, more cost-effective solutions should be sought. Such solutions can be systems based on 2D scanners. The available 2D scanners have a range of up to several meters and are many times cheaper.

When using 2D scanners for room mapping, a mechanism may be needed to ensure their movement. Zhang et al. presented a solution where a servo mechanism was used to rotate the Hokuyo scanner by an angle between −90 and 90 degrees [25]. On the other hand, Surmann et al. presented the use of a laser rangefinder mounted on a rotating base driven by a servo motor, which provided rotation in the horizontal axis [26]. However, it should be noted that these solutions are not sufficient to ensure the safety of the wheelchair user. The use of a rotary mechanism and a 2D LiDAR scanner can allow the 3D mapping of the environment for a wheelchair anti-collision system. Therefore, this paper presents a proprietary solution of a rotational mechanism for a 2D scanner that enables 3D scene mapping around a vehicle. The proposed scanning system consists of three of these units. The model of the environment is built sequentially by assembling data obtained from different angles. The appropriate arrangement of the developed units on the wheelchair ensures 360-degree observation of the surroundings at various heights, which is essential for full protection of the person in the wheelchair.

## 2. Problem Statements

People with disabilities often have limited ability to control the wheelchair in response to emerging threats. Changing elements of the environment, such as the appearance of new objects that may pose a threat, can also be a significant problem. Additionally, depending on the type of disability, people with disabilities may have limited ability to observe their surroundings. Therefore, there is a risk of collision that can be dangerous for the wheelchair user, as well as for other people.

Even small transverse obstacles can pose a danger to people with disabilities. Therefore, a system that detects them is needed. The obstacles around the wheelchair can be at different heights. Therefore, according to the authors, it is necessary to map the environment so that hazards can be detected. The construction of the map requires distance sensors that guarantee high resolution and accuracy. Ultrasonic sensors, although cheap, do not allow one to obtain sufficiently accurate maps of the environment. Therefore, laser scanners were chosen to develop the system.

### Research Goals

Safety should be a key factor in the development or improvement of wheelchairs. Therefore, the main objective of this study was to develop a mechatronic anti-collision system for wheelchairs based on 2D LiDAR laser scanners. The design of this system was guided by the following assumptions:

- The solution should not be complicated to operate; therefore, the system should be semiautomatic.
- Obstacles in the wheelchair environment can be at different heights; therefore, the system should provide information about the 360-degree space from the ground height up to at least the head height.
- The solution should be of a small size, as the structural volume of the wheelchair available for the installation of additional elements is limited.
- Positioning of the individual elements of the system must not require additional operations, so these elements must be permanently fixed to the wheelchair.
- The cost of the developed solution should be significantly lower than that of complex and expensive automation systems in vehicles for the disabled.

Considering these assumptions will improve the quality of life of people with disabilities by increasing their mobility, independence, and level of social involvement while respecting their needs and preferences.

## 3. Design of the Scanning Mechanism

**Figure 1:**
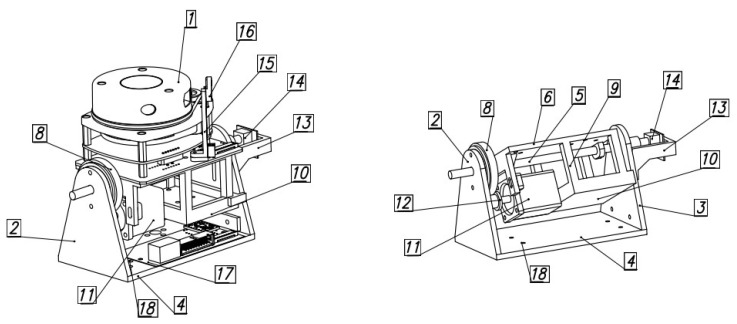
Scheme of the scanning mechanism: 1—scanner, 2—right bracket, 3—left bracket, 4—base, 5—shaft, 6—platform, 7—first fastening hole, 8—idler gear, 9—support, 10—shelf, 11—motor, 12—movable gear, 13—first arm, 14—rotation sensor, 15—second arm, 16—detector, 17—electrical board, 18—second fastening hole.

The RPLidar scanning system allows for scanning by performing a rotation about a single axis. This solution is insufficient when a larger range of data must be acquired. For this reason, a mechanism was designed that allows the entire scanning module to rotate around the second axis (Figure 1). The mechanism consists of an RPLidar scanner placed on a platform rigidly connected to the rotary axis. The platform is equipped with a stepper motor with a gear mounted on it, which cooperates with a second wheel fixed in the base. This configuration allows the platform to rotate relative to the base. Therefore, the platform has a weight pocket. Adequate loading of the platform (balancing of the system) ensures that the torque required to rotate the scanner is reduced and that it returns spontaneously to its rest position. Since high positioning accuracy is required and the helical gear cannot provide it due to circumferential backlash, the end of the rotary axis is connected to a potentiometer to determine the exact swing of the platform. The scanning planes during the operation adopt a helical character.

The accuracy of the device depends on its design and the applied stepper motor and gearbox. The completed model provides a tilt-angle change with a resolution of 0.45°. Since the scene is built sequentially, decreasing the step increases the detail but also increases the time to build the scene. The angular resolution should be a compromise between the speed of reproducing the whole scene and its detail.

**Figure 2:**
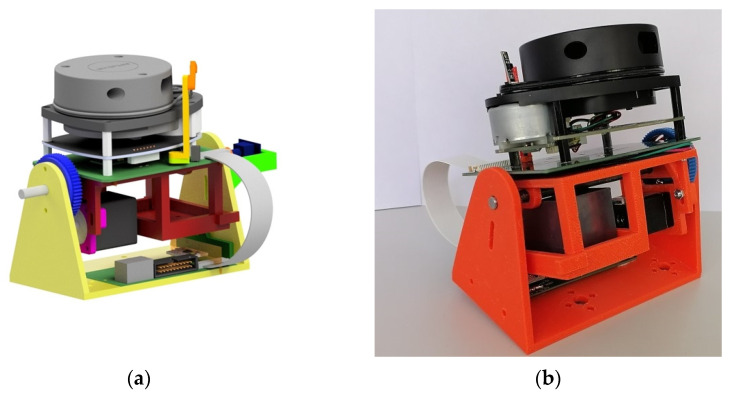
(**a**) Visualization of the scanning mechanism; (**b**) prototype of the scanning mechanism manufactured by 3D printing.

In order to produce the prototype of the scanning mechanism, its individual parts were made using the additive manufacturing (AM) method. AM is defined in the international standard ISO/ASTM 52,900 as a “process of joining materials to make parts from 3D model data, usually layer upon layer”. AM processes can be divided into seven categories: material extrusion, material jetting, binder jetting, powder bed fusion, directed energy deposition, vat polymerization, and sheet lamination [27]. For this particular application, the process of material extrusion known as fused deposition modeling (FDM) was chosen. This process consists of the extrusion and subsequent deposition of a molten filament of a polymeric material. Compared to other 3D-printing techniques, FDM is known for its high printing speed and low cost [28,29]. The individual parts were printed from acrylonitrile–butadiene–styrene (ABS), which is a thermoplastic material. The printed parts were assembled, and the prototype device is shown in Figure 2b.

The scanner positioning mechanism designed in this manner has been filed for patent protection in Poland as: “Urządzenie do ustawiania położenia skanera z wykorzystaniem tego urządzenia oraz zastosowanie urzędzenia do ustawiania położenia skanera [A scanner positioning device, a method for positioning a scanner using the device, and an application of the scanner positioning device]” under the number P.437266. However, the presented prototype of the mechanism that sets the position of the scanner is not the final solution but only provides the implementation of the tilting movement. Further work will attempt to reduce the size of the device as well as develop its case.

## 4. Control System of the Scanning Mechanism

**Figure 3:**
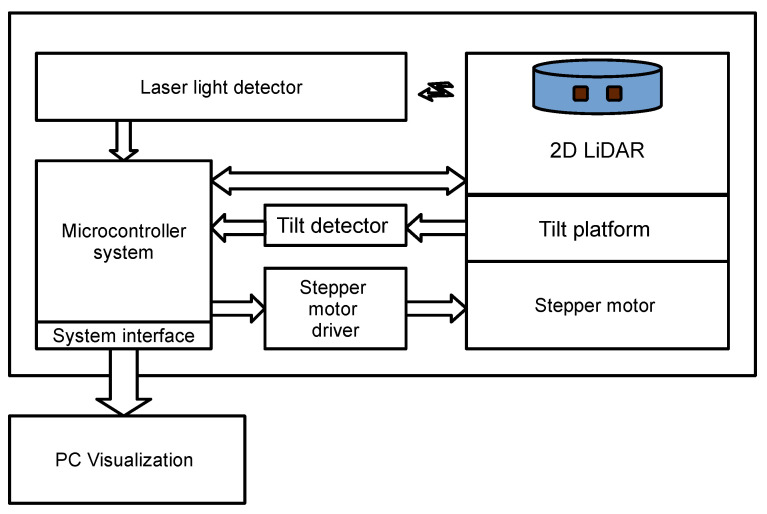
Block diagram of the scanning mechanism control system.

The scanning system uses an RPLIDAR A1 scanner that communicates with the microcontroller through a UART interface. The laser beam detector is constructed from a photodiode and an analog voltage comparator, and the output of the comparator is connected to the interrupt input of the microcontroller. The platform is driven by a stepper motor, which is controlled directly from the microcontroller. The angular resolution of the motor is 1.8°, while the gearbox provides a change in the tilt angle of the platform with a step of 0.45°. The measurement of this angle is performed by a rotary potentiometer that is mechanically coupled to the rotating platform. The necessary number of steps to reach a new scanning plane is determined in the microprocessor system before the rotation begins. Rotation is initiated when laser light is detected by the detector. The detector does not change its position relative to the LiDAR but is tilted along with it and the platform. A laser pulse is generated when the rotating scanner passes through the position where the detector is mounted. The location of the detector mount determines the angle of the scanner coordinates relative to its zero position. This location can be arbitrary but is determined when the detector is mounted. Turning on the wheelchair activates the system, which then starts scanning. The block diagram of the scanning mechanism control system is shown in Figure 3.

The rotary potentiometer has a range of motion of 300° and a resistance of 1 k, and it is powered by 5 V. The potentiometer signal is measured by an ADC with 12-bit resolution, which gives an angle measurement resolution of about 0.07°. In future solutions, it is planned to use an inertial measurement unit (IMU) to determine the tilt angle of the platform. As a result, it is expected that the actual tilt angle will be obtained independently of the slope of the terrain, which should improve the quality of the obtained image.

## 5. Data Acquisition

**Figure 4:**
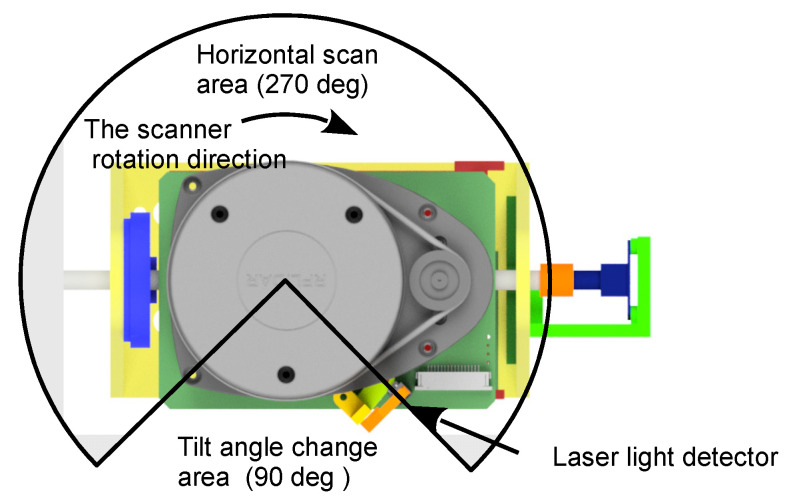
Schematic showing the scanning area, including scanner rotation and laser light detector placement.

The designed mechanism was evaluated under conditions similar to real ones. The experiments used a step change in the tilt angle of the platform and were performed over a range of scanning angles in which the scanned area is obscured by the structure of the wheelchair and is not usable. In this mode of operation, the maximum allowable range of a tilt change in one step depends on the speed of the scanner, the torque of the engine, and the range of unusable angles. The ranges for scanners placed at the front and rear of the carriage are 90° and 180°, respectively. The effective ranges of scanning angles in this case are 270° and 180° in the horizontal plane, respectively. In an area covering a 90-degree angle (tilt-angle change area), there may be a change in the angle of the scanner outside the measurement area (Figure 4). This results in a constant scanning plane over a useful range of angles.

The detector location must be selected so that the generated pulse falls at the beginning of the zone where the scanning area is obscured. The same pulse is used to assign a timestamp at which the scanner reaches a particular position. On the basis of the speed of the scanner, the execution time of each measurement point is determined. The system can operate in continuous angle change mode when a full 360-degree horizontal scanning range is required. In that case, the scanning planes assume a helical character, and the detected light signal is used to synchronize the data with the tilt angle.

The applied LiDAR scanners are small-range solutions with a sampling rate of 4000 samples/s. During the mapping, part of the scanner’s field of view is obscured by the cart and the user. Such data are discarded, which greatly facilitates the processing of the collected data.

To control the system, a MyRiO unit equipped with a dual-core microprocessor running at 533 MHz was used. However, this unit was not sufficient to perform the calculations, so they were performed on the computer. The MyRiO unit, on the other hand, acted as the measurement and execution system. A suitable computing unit will be selected in further research work.

### 5.1. Theoretical Calculations

The motor torque required to rotate the platform depends on the required rotation speed and the inertia of the system. The torque on the axis of rotation *M*’ is expressed by Equation (1):

(1) $$M^{\prime }=\frac{J_{z}d\omega_{1}}{dt}$$

where *J_z_* is the moment of inertia of the system in the direction of rotation, and $d\omega_{1}$ is the change in rotational speed.

The moment of inertia depends on the mass and its distribution. In the prototype model, the mass of the LiDAR used is concentrated around the spinning head, the motor that drives it, the stepper motor, and the mass of an additional weight. Assuming a simplified model of such a system in the form of a homogeneous cylinder for the head and the motor and a cubic mass system for the stepper motor and the weight, the moment of inertia is expressed by Equation (2):

(2) $$\begin{array}{c} J_{z}=m_{1}\left(\frac{1}{4}R_{1}^{2}+\frac{1}{12}b_{1}^{2}+h_{1}^{2}\right)+m_{2}\left(\frac{1}{4}R_{2}^{2}+\frac{1}{12}b_{2}^{2}+h_{2}^{2}\right)\\ +\;m_{3}\left(\frac{1}{12}\left(b_{3}^{2}+c_{3}^{2}\right)+h_{3}^{2}\right)+m_{4}\left(\frac{1}{12}\left(b_{4}^{2}+c_{4}^{2}\right)+h_{4}^{2}\right), \end{array}$$

where *m*_1_ is mass, *R*_1_ is radius, *b*_1_ is height, and *h*_1_ is the distance of the center of mass from the axis of rotation of the spinning head. The designations with index 2 refer to the motor driving head. While *m*_3_ is the mass of the motor, *b*_3_ and *c*_3_ are the dimensions of the motor perpendicular to the pivot axis of the platform, which are, respectively, the width and height (ignoring the length of the motor), and *h*_3_ is the distance from the pivot axis to the center of mass of the motor. The symbols with index 3 refer to the weight.

The moment on the motor axis *M* is expressed by Equation (3):

(3) $$M=\frac{r_{1}}{r_{2}}M^{\prime }$$

where *r*_1_ and *r*_2_ are, respectively, the radii of the motor and platform gears.

The numerical relationships for the three selected values of the change in tilt angle in 1 min and for five different scanner rotation speeds $\omega_{2}$, with the moment of inertia of the system under test *J_z_* = 0.0016 kg m^2^, are shown in Table 1, where *t*_90_ is the time that it takes for the scanner to make a 90-degree rotation, $\omega_{1min}$ is the minimum platform rotation speed necessary to obtain a new scanner position in a given time, and *M*_min_ is the minimum torque on the motor shaft necessary to obtain the minimum rotation speed.

The estimation of the necessary torque is intended to facilitate the selection of a motor capable of turning the platform in a sufficiently short time. This is an approximate solution that does not take into account factors such as friction, gear efficiency, or the mass of other components, considering their influence to be minor. Taking these aspects into account, the engine should have a torque reserve.

Achieving a fast pitch rotation of the platform is challenging because it requires significant torque to achieve sufficient acceleration when starting and stopping. The approximate time to rotate the platform at a given angle α, assuming that the motion is uniformly accelerated for the first half and uniformly decelerated for the second half of the scanner rotation time around the tilt axis, is described by relation (4):

(4) $$t=\sqrt{\frac{4\alpha \cdot J_{z}}{\frac{r_{2}}{r_{2}}M}}$$

The numerical values for the selected values of angle α and a motor with a torque of 0.06 Nm are shown in Table 2.

### 5.2. Experimental Investigation

The drive of the prototype platform is a stepper motor with a torque of 0.06 Nm. Based on the calculations in Table 2, it appears that a motor with this torque should provide a 5° tilt-angle change in 21 ms for all available scanner speeds. Stepper motors are characterized by so-called “losing steps”. This phenomenon occurs when the motor is overloaded, for example, in the initial phase of movement. For this reason, the speed of the motor that drives the platform is not constant, and the duration of the steps is variable. During the initial and final phases of the movement, when acceleration and deceleration occur, the duration of the steps is increased.

**Figure 5:**
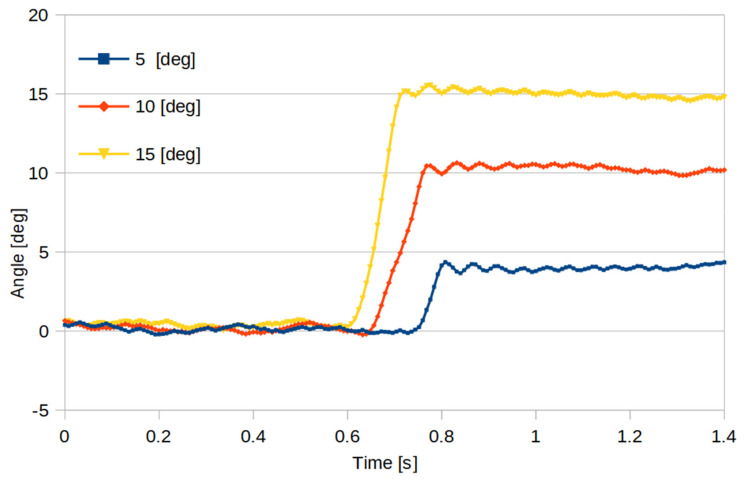
Time of change in position for angles as a function of time.

To evaluate the performance of the mechanism, an experimental study was conducted. The behavior of the motor was checked at the selected angle values. The results of the experimental tests are shown in Figure 5.

## 6. Results and Discussion

The results confirmed the theoretical data. Assuming that the horizontal non-scanning zone is 90°, and the rotation speed does not exceed 10 r/s, it can be concluded that the prototype model allows one to obtain a change in the tilt angle of the platform by a maximum of 5° for each rotation of the scanner. For lower scanner rotation speeds, the range of angle change can be larger. The results show that the same effect can be obtained by increasing the non-scanning zone or by increasing the motor torque.

**Figure 6:**
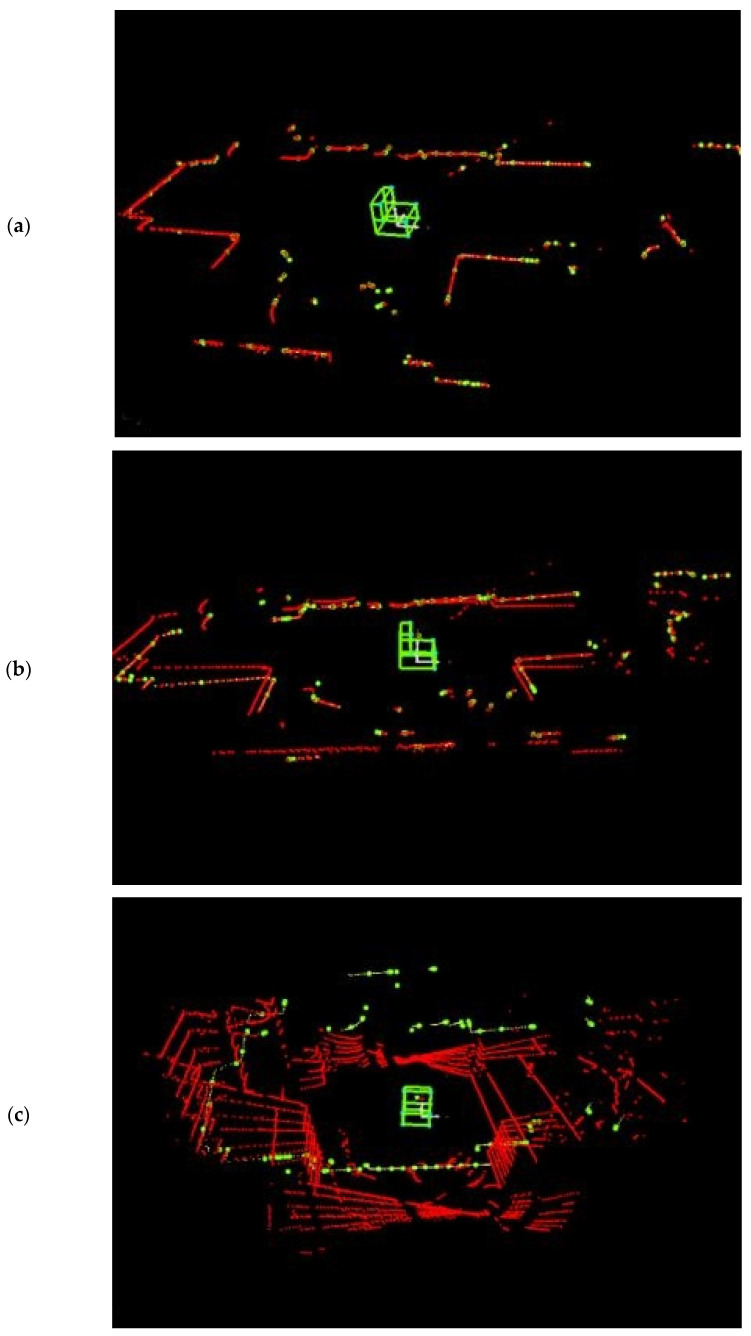

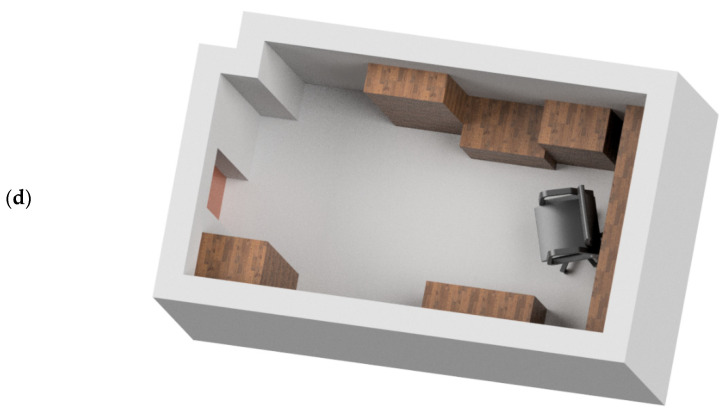
Map construction stages: (**a**) the first scan to create a map, (**b**) the second scan added to the map, (**c**) the map created after submitting several scans, and (d) visualization of the mapped room.

The presented solution provides a 3D image of the environment in a wide range of vertical angles from −45° to 90°. This image is built sequentially by adding successive scans taken in different scanning planes. Figure 6 shows the stages of map construction in the scanning angle stepping mode. The scanner is located where a solid representing a simplified wheelchair model is visible. The axis of the scanner tilt mechanism is parallel to the direction of travel. Figure 6a shows the initial stage of map construction. The first scan is marked in red. Figure 6b shows the next stage with two scans taken in scanning planes tilted 0 and 5° from the horizontal. Figure 6c shows a map constructed from seven scans taken from the same position and with the same scanner orientation.

The ability to observe the environment on the horizontal scanner axis is limited. However, this is not a problem in mobile applications. When changing position, the area with limited visibility is automatically filled with measurements. The same situation occurs when changing orientation. In Figure 6c, a scan taken when the orientation changes by about 90° is marked in yellow.

**Figure 7:**
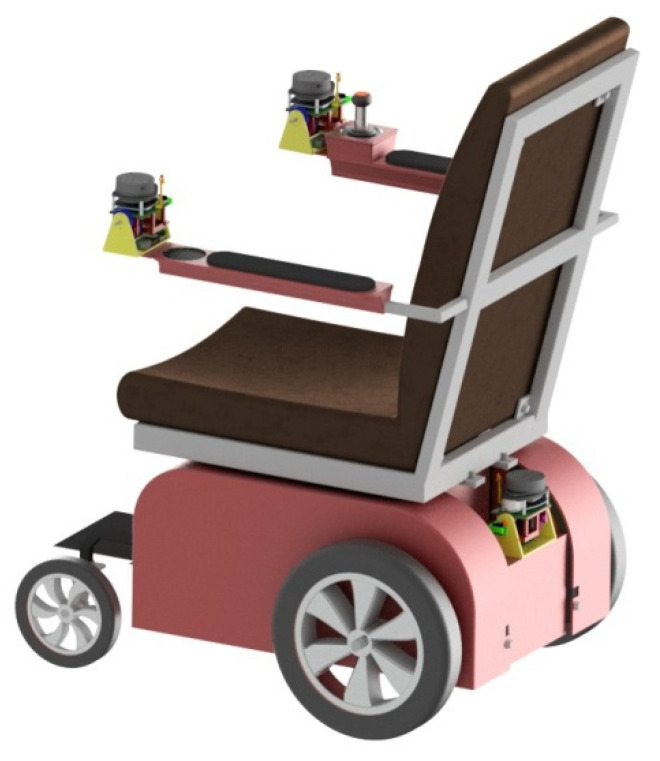
Arrangement of scanning devices in the wheelchair.

A single scanning device is not a complete solution because it does not provide sufficient coverage of the scanned area. For this, a set of several scanners is necessary. It turns out that three sensors are the minimum for this purpose. One of them will be placed at the back of the cart, while the other two will be placed on both armrests, as shown in Figure 7.

**Figure 8:**
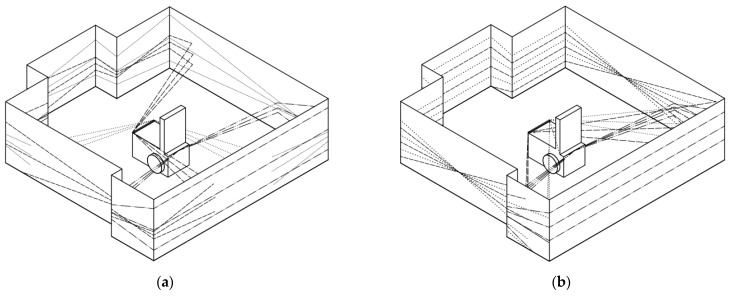
Scanning area for axes of scanning devices positioned: (**a**) in one line and (**b**) not parallel.

When the sensors are placed at the front so that the rotation axes of the scanner positioning mechanisms are colinear, peculiar areas appear. These areas are excluded from the scanning area, as shown in Figure 8a. These areas can be nullified by positioning the sensors so that their rotation axes are not parallel. The scanning area with this sensor placement is shown in Figure 8b. The use of three scanning devices makes it possible to make a map of the environment around the cart, regardless of the direction in which the wheelchair is moving.

The basis for such a scanner configuration was the assumption that the system should be convenient for disabled people. At the same time, the possibility of folding and unfolding the wheelchair for transport must be preserved. For this reason, the elements of the system must not require additional operations to be set up and, therefore, must be permanently attached to the wheelchair.

Placing LiDAR scanners above the user’s head could make it possible to capture an image all around the chair. However, this placement of the scanner is not practical because to fold the wheelchair, the scanner would have to be mounted on an extension and could require additional steps to set up.

### Limitations and Further Studies

The presented solution is one of the elements of the designed wheelchair security system. The solution cannot be disclosed in full because it will be filed for patent protection. The collision avoidance system under development is based on mapping objects around the wheelchair. Further work should include map construction and SLAM issues. Based on the work in [15,30], proprietary algorithms are being developed: the odometry-based localization algorithm and the scan matching algorithm (ICP). The algorithms being developed are not described in the presented work because they are part of a solution that will be filed for patent protection and therefore cannot be disclosed.

Currently, the selected control unit is not sufficient to perform the calculations. Therefore, further work is required, which will result in the selection of a suitable unit.

The wheelchair, as a human-guided device, can move at any time during measurements. This could lead to distortions in the image obtained from the scanner. This problem was discussed in the study by Henawy et al. [31], where, in order to correct the motion distortions for LiDAR inertial odometry (LIO), the authors proposed an accurate piecewise linear deskewing algorithm using high-frequency motion information provided by an inertial measurement unit (IMU). The authors’ approach to this problem is presented in [32].

## 7. Conclusions

Experimental studies and numerical analysis were performed to verify the correctness of the assumption. On the basis of this research, the validity of the assumption was demonstrated. The rotating mechanism enables 3D scene acquisition using a cost-effective 2D scanner system. The proposed proprietary security system can be used in wheelchairs and electric vehicles for the disabled.

Importantly, we found that an essential issue is the arrangement of the scanning system elements in the structural volume of the wheelchair. This is important for a proper representation of a 360-degree scene around a wheelchair while maintaining its full functionality, i.e., moving, boarding, and dismounting a disabled person, and enabling its transportation. The proposed solution does not limit these functionalities but significantly improves the safety of people in wheelchairs. Furthermore, the miniaturization of the individual parts of the system and the integration of the safety systems into the structural system of the wheelchair will have a positive impact on its ergonomics and ease of use.

## Figures and Tables

**Table 1:** Results of numerical calculations.

$\omega_{2}\;(\mathrm{rpm})$	*t*_90_ (s)	$\omega_{1\min}$			*M*_min_ (Nm)		
		1°	5°	10°	1°	5°	10°
5	0.050	0.349	1.744	3.489	0.00014	0.00065	0.00133
6	0.042	0.419	2.093	4.187	0.00016	0.00080	0.00160
7	0.036	0.488	2.442	4.884	0.00019	0.00093	0.00187
8	0.031	0.558	2.791	5.582	0.00021	0.00106	0.00213
9	0.028	0.628	3.140	6.280	0.00024	0.00119	0.00240
10	0.025	0.698	3.489	6.978	0.00027	0.00133	0.00266

**Table 2:** Rotation time of the platform for selected angles.

α (°)	*t* (s)
1	0.010
2	0.014
5	0.021
10	0.030
15	0.037

## Data Availability

The data presented in this study are available on request from the corresponding author.
